# miR-139-5p modulates cortical neuronal migration by targeting Lis1 in a rat model of focal cortical dysplasia

**DOI:** 10.3892/ijmm.2014.1703

**Published:** 2014-03-18

**Authors:** YANJUN HUANG, JIAO JIANG, GUO ZHENG, JING CHEN, HAIYING LU, HU GUO, CHUNFENG WU

**Affiliations:** Department of Neurology, Nanjing Children’s Hospital, Nanjing Medical University, Nanjing, Jiangsu 210008, P.R. China

**Keywords:** microRNA, miR-139-5p, Lis1, neuronal migration

## Abstract

Accumulating evidence has indicated that microRNAs (miRNAs or miRs) play important roles in the developing rat brain. In this study, we investigated the role of miRNAs in the brains of immature (20–80 days) rats with liquid nitrogen lesion-induced focal cortical dysplasia. miRNA microarray demonstrated that the expression of miR-139-5p was associated with cortical development. Bioinformatic analysis and luciferase assays revealed that the *Lis1* gene is a likely target of miR-139-5p. It is known that *Lis1* plays a role in cell proliferation and migration and can lead to cortical dysplasia when mutated. Our data demonstrated an inhibitory effect of miR-139-5p on the expression of *Lis1* in PC12 cells 24 h following transfection with pre-miR-139-5p. However, when the PC12 cells were transfected with anti-miR-139-5p, an increase was observed in the expression of *Lis1*. Cell migration assay revealed that miR-139-5p significantly inhibited the migration of PC12 and HCN-2 cells treated with or without Lis1 protein. In addition, a rat model of focal cortical dysplasia was established, wherein miR-139-5p was administered and *Lis1* expression was found to be markedly reduced. Moreover, the injured cortex showed a certain degree of recovery following the administration of miR-139-5p, demonstrating that the reduction in miR-139-5p was at least partially responsible for the upregulation of *Lis1* in the rat brains. Our data suggest that miR-139-5p modulates cortical neuronal migration by targeting *Lis1*.

## Introduction

Cell proliferation, neuronal migration and cortical organization represent three important stages of brain maturation (1). Cortical development is critical for brain maturation as cortical malformations are increasingly recognized as causes of severe epileptic syndrome, neuropsychological disorders and mental retardation (2,3). Moreover, the incorrect positioning of cortical neurons following cortical cell migration often leads to cortical dysplasia. Therefore, the identification of regulatory factors responsible for brain development and maturation is important for understanding the pathogenesis of cortical abnormalities.

*Lis1* is a brain-specific gene that encodes for the non-catalytic subunit of platelet-activating factor acetylhydrolase isoform 1B (PAFAH1B), which inactivates platelet-activating factor (PAF) (4,5). *Lis1* is known to regulate cell proliferation and migration during brain development through its interaction with proteins, such as dynein (6). Subjects with Miller-Dieker syndrome (MDS) or isolated lissencephaly sequence (ILS) have a hemizygous deletion or mutation of the *Lis1* gene (7,8). ILS and MDS often result from haploinsufficiency at human chromosome 17p13.3, a chromosomal region that includes the *Lis1* gene. The disruption of *Lis1* in patients with ILS and MDS (9) suggests that mutations within *Lis1* are responsible for defective neuronal migration.

microRNAs (miRNAs or miRs) are a class of small, non-coding, regulatory RNA molecules (10). Over the past decade, research has identified important regulatory roles for miRNAs in cell development, differentiation, proliferation, apoptosis and metabolism, as well as in the pathogenesis of several diseases (11). Approximately 70% of all known miRNAs are expressed in the mammalian brain, and the levels of many miRNAs are dramatically altered during brain development (12). However, the roles of miRNAs in the regulation of mammalian brain development are still poorly defined (13).

Further knowledge of the molecular mechanisms underlying cortical neuronal migration may provide insight into improved therapeutic options for the treatment of malformations of cortical development. In this study, we examined miRNA expression profiles in immature rats with liquid nitrogen lesion-induced focal cortical dysplasia. Our aim was to identify the miRNAs that modulate cortical neuronal migration. We identified and characterized miR-139-5p, indicating that the loss of miR-139-5p regulates cortical neuronal migration through the modulation of *Lis1* expression.

## Materials and methods

### miRNA microarray analysis

RNA labeling and hybridization to miRNA microarray chips were performed as previously described (14). Whole brain tissues from immature Sprague-Dawley (20–80 days) rats were pooled and total RNA was extracted using TRIzol (Invitrogen, Shanghai, China). Briefly, 50 mg of total RNA were purified using the mirVana miRNA isolation kit (Ambion, Austin, TX, USA) resulting in a small, enriched RNA fraction. Purified RNA was labeled with Cy3 and hybridization was carried out using a miRNA microarray chip (CapitalBio Corp., Beijing, China) containing 381 probes in triplicate.

### Quantitative reverse transcription-polymerase chain reaction (qRT-PCR) of miR-139-5p

We performed miRNA qRT-PCR as previously described (15). Briefly, rat brain RNA (1 μg) was reverse transcribed with a stem-loop reverse transcriptase primer and quantitative PCR (qPCR) was then performed. The program was initially 2 min at 95°C, followed by 40 cycles of 30 sec at 95°C, and 60 sec at 60°C. The primers used for miR-139-5p qRT-PCR were as follows: stem-loop RT primer, 5′-CTCAACTGGTGTCGTGGAGTCGGCAATTCAGTTG AGAGACACGT-3′; and the qPCR primers: miR-139-5p forward, 5′-ACACTCCAGCTGGGTCTACAGTGCAC-3′ and reverse, 5′-TGGTGTCGTGGAGTCG-3; and U6 forward, 5′-GCTTC GGCAGCACATATACTAAAAT-3′ and reverse, 5′-CGCTT CACGAATTTGCGTGTCAT-3′.

### Analysis of miR-139-5p predicted targets

The prediction of miR-139-5p targets was performed using the following algorithms: PicTar (http://pictar.mdc-berlin.de/), TargetScan, (http://www.targetscan.org/vert_50/) and miRanda (http://www.ebi.ac.uk/enright-srv/microcosm/cgi-bin/targets/v5/mirna.pl).

### Cell culture and transfection

PC12 cells were maintained in DMEM high glucose medium, supplemented with 10% fetal bovine serum (FBS) (both from Gibco, Carlsbad, CA, USA) and 5% horse serum. The cells were cultured in a humidified incubator at 37°C with 5% CO_2_. The PC12 cells were transfected with 50 nM of either a non-targeting small RNA oligonucleotide (GenePharma Co., Ltd., Shanghai, China) as a negative control (control), or miR-139-5p mimics (stably enhanced miR-139-5p oligonucleotide; GenePharma Co., Ltd.) using Lipofectamine™ 2000 (Invitrogen, Carlsbad, CA, USA), as previously described (16). The transfected cells were harvested after 48 h, RNA was extracted from the cells using TRIzol reagent (Invitrogen, Shanghai, China), and proteins were extracted using lysis buffer [20 mM Tris (pH 7.4), 1 mM EDTA, 1% Triton X-100, protease inhibitors] (GE Healthcare Life Sciences, Shanghai, China), as previously described (17). The subsequent qRT-PCR and western blot analysis results were obtained from three separate transfections.

### qRT-PCR of Lis1

The mRNA qRT-PCR analyses were conducted as previously described (18). Briefly, RNA (1 μg) obtained from the rat brains or PC12 cells was reverse transcribed using oligo(dT)18 followed by qPCR. The program was initially run for 2 min at 95°C, followed by 40 cycles of 30 sec at 95°C, and 60 sec at 60°C. The primers used for Lis1 qRT-PCR were as follows: Lis1 forward, 5′-TGCCCAAGACTACTCAACCC-3′ and reverse, 5′-GCACCCTGTGACGAAAGC-3′; and 18S RNA, forward, 5′-AGCAACTGCGCCTGAAAC-3′ and reverse, 5′-CCCTGTCCCGCTCAACTA-3′.

### Western blot analysis for Lis1

The cells were rinsed once with phosphate-buffered saline (PBS) then lysed in lysis buffer [50 mmol/l Tris-HCl (pH 7.5), 5 mmol/l EDTA, 1% (V/V) Triton X-100 and 0.15 mol/l NaCl] on ice for 10 min. Insoluble components were removed by centrifugation (12,000 rpm for 5 min), and the protein concentration was measured, as previously described (19). Cellular proteins (100 μg) were adjusted to a total concentration of 5 μg protein/μl. After boiling for 5 min in loading buffer, proteins were separated by 8% Tris-glycine gels for Lis1. Western blot analysis was performed using mouse anti-rat Lis1 antibody (Abcam Biotech, Cambridge, MA, USA) and goat anti-mouse HRP-linked secondary antibody. Immunocomplexes were visualized using the LumiGLO^®^ chemiluminescent detection kit (Cell Signaling Technology, Boston, MA, USA) according to the manufacturer’s instructions.

### Luciferase targeting assay and Transwell assay

We cloned 400 bp of the Lis1 3′ untranslated region (3′UTR) containing the 7-bp target site for miR-139-5p into the *Spe*I/*Hin*dIII sites of a luciferase gene in the pMIR-REPORT luciferase vector (Ambion, Shanghai, China). PCR analyses were performed using rTaq polymerase (Takara Bio Inc., Japan). The PC12 cells (5×10^4^) were co-transfected with 150 ng of pMIR-REPORT, Lis1-3′UTR plasmid and 25 nM of either a stably-enhanced non-targeting small RNA oligonucleotide as a negative control (control), or miR-139-5p mimics (both from GenePharma Co., Ltd.) using Lipofectamine™ 2000 (Invitrogen, Shanghai, China), as previously described (20,21). Cells transfected with pre-scramble miRNA or or anti-scramble miRNA were also used as negative controls. The transfected cells were harvested after 24 h and then assayed using a Dual-Luciferase Reporter assay system (Promega, Madison, WI, USA). The results were obtained from three separate experiments with each one conducted in triplicate.

A Transwell migration assay was performed as previously described (22). The cells (2×10^5^) were seeded onto a 35-mm dish one day prior to transfection. The transfection protocol was conducted according to the cell culture and transfection protocols previously described (23). After 24 h of transfection, 5 ng/ml of Lis1 protein were added to the cell culture medium. The PC12 and HCN-2 cells that had migrated onto the membrane were fixed with methanol and stained with crystal violet after 24 h. Images of randomly selected fields of the fixed cells were acquired and the cells were counted. Experiments were repeated in triplicate.

### Rat model of focal cortical dysplasia

Thirty-three days after birth, 30 Sprague-Dawley rats were obtained from the Laboratory Animal Center of Nanjing University (Nanjing, China). All animals were housed in a specific pathogen-free (SPF) facility and received human care according to the Chinese legal requirements. The experimental animal procedures were approved by the Nanjing Medical University Institutional Animal Care and Use Committee. All experiments were performed according to the guidelines of the European Community Council.

The rat model of liquid nitrogen lesion-induced focal cortical dysplasia was established as previously described (24). Twenty Sprague-Dawley rats with cortical dysplasia were randomly divided into two groups: the model group and the miR-139-5p administration group. In the model group, brain tissue was obtained on days 0, 20, 60 and 80. In the miR-139-5p administration group, miR-139-5p was injected into the rat brains using a rat brain locator at a 5 mg/kg dose on days 40 and 50, and brain tissue was obtained on day 60. The control (healthy; n=10) group was administered the same volume of PBS. On day 60, brain tissue was rapidly removed and prepared for hematoxylin and eosin (H&E) and Nissl staining.

### H&E and Nissl staining

The rat brain tissues were rapidly fixed in 10% formaldehyde and embedded in paraffin. Sections (4-μm-thick) were mounted for H&E and Nissl staining. Brain tissue morphological characteristics and differences between the three experimental groups were observed under a microscope (Nikon, Tokyo, Japan).

### Statistical analysis

All experiments were performed in triplicate. Hierarchical cluster analysis was carried out using Gene Cluster software (Stanford University, Stanford, CA, USA). Comparisons between groups were made by a Student’s t-test. A P-value <0.05 was considered to indicate a statistically significant difference, as previously described (25).

## Results

### Cluster analysis for microarray data and qRT-PCR validation

**Figure 1:**
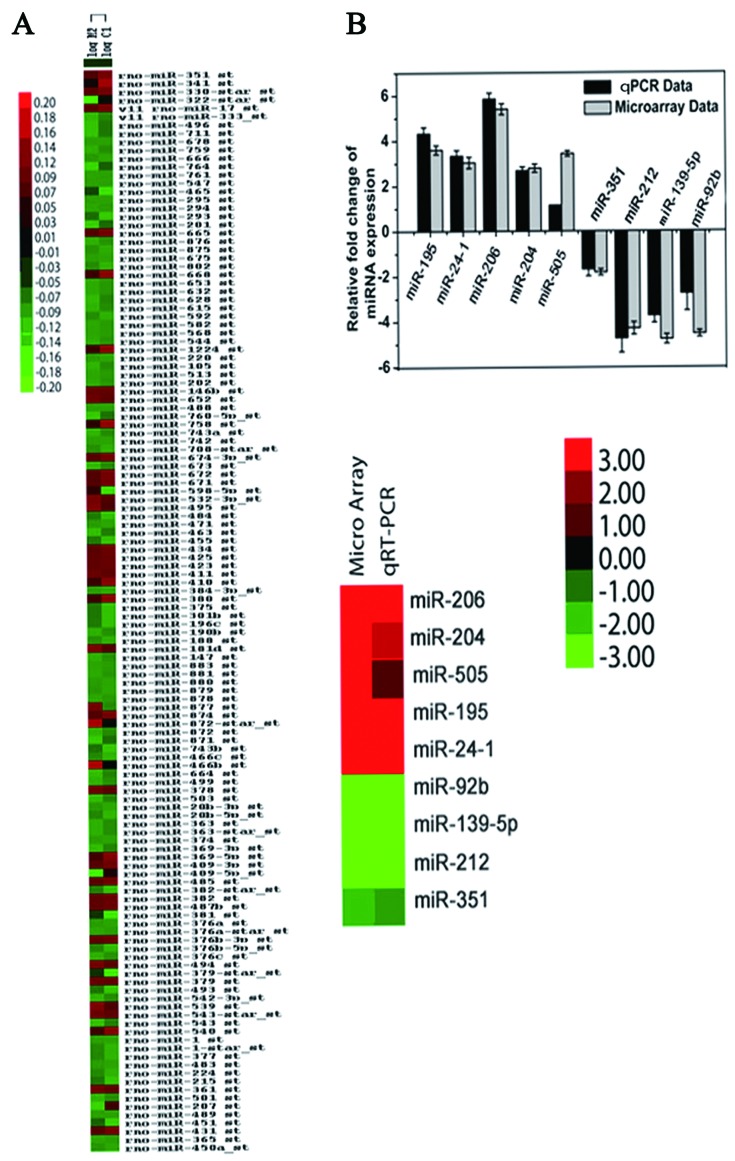
Cluster analysis of aberrant microRNA (miRNA) expression in immature rats with focal cortical dysplasia of induced by liquid nitrogen lesions and assayed by microarray and qRT-PCR. (A) Dendrogram generated by cluster analysis showing the separation of the dysplasia model from the control samples on the basis of miRNA profiling. (B) The relative fold changes of miRNAs from qRT-PCR data compared with microarray data.

We examined the expression profiles of miRNAs in immature rats with liquid nitrogen lesion-induced focal cortical dysplasia. The dendrogram generated by cluster analysis revealed the separation of the model of dysplasia from the control samples on the basis of miRNA profiling (Fig. 1A). The relative changes in miRNA expression as shown by the qRT-PCR data were in agreement with the microarray data (Fig. 1B). These results indicate a downregulation in miR-139-5p expression during rat brain development.

### miR-139-5p targets Lis1 mRNA as shown by luciferase assay

**Figure 2:**
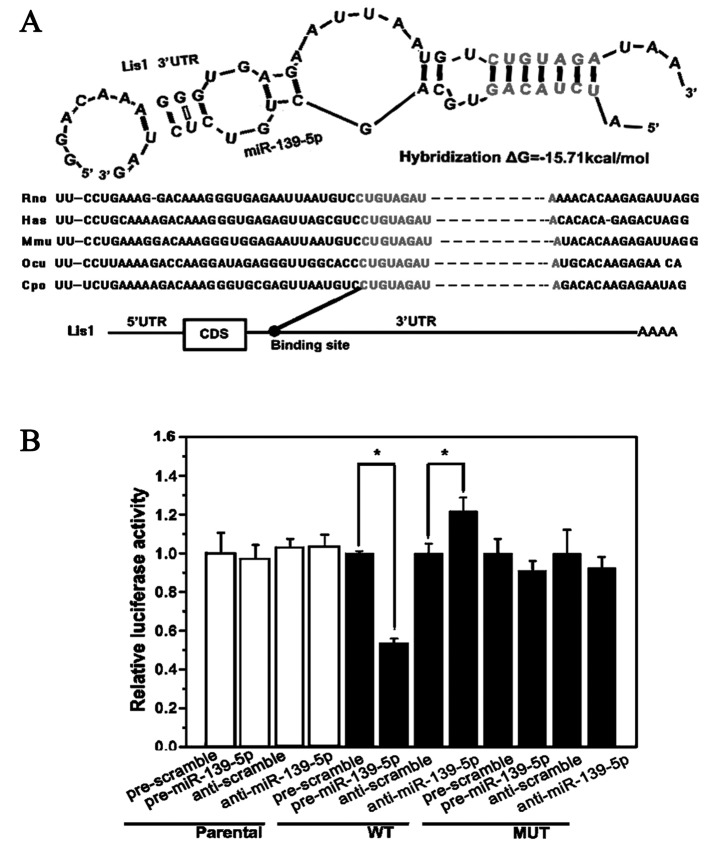
Predicted targets for miR-139-5p. (A) Schematic description of conserved binding sites for miR-139-5p. The seed-recognition site was demarked and all nucleotides in this region were completely conserved among several species. Hypothesized duplexes formed by interacting between the binding sites of *Lis1* 3′UTR (top panel) and miR-139-5p (bottom panel) were illustrated, and the predicted free energy of each hybrid was indicated. (B) Analysis of luciferase activity in the PC12 cells transfected with pre-miR-139-5p or anti-miR-139-5p. PC12 cells were co-transfected with a pre-miR-139-5p or anti-miR-139-5p, luciferase reporter plasmid containing the wild-type (WT) or mutant (MUT) 3′UTR of *Lis1* mRNA and β-gal as the control plasmid. Data are presented as the means ± standard deviation (SD); ^*^P<0.05. 3′UTR, 3′ untranslated region.

Multiple algorithms (TargetScan, miRanda and PicTar) for predicting the putative targets of miR-139-5p identified several potential targets, such as *Lis1*, *Capn8*, *Gmfb*, *Mapk1*, *Dclk1*, *Vim*, *Mgst1*, *Gnb1*, *Klf15* and *Cdkn1b. Lis1* was selected for further examination due to its established role in cell proliferation and migration, as well as its role in brain development (26). Bioinformatic analysis for the target site of miR-139-5p in the *Lis1* 3′UTR revealed that mature miR-139-5p shares the same sequence in rats, mice and humans (Fig. 2A). There is one 7 bp target site in the *Lis1* 3′UTR which is conserved in mammals. Wild-type or mutant *Lis1* 3′UTR was constructed in the luciferase reporter plasmid for conducting luciferase targeting assays (Fig. 2B) as it contains the 7-bp target site for miR-139-5p and the mutant 7-bp site. The luciferase activity of the wild-type plasmid was suppressed by approximately 42% at 24 h in the PC12 cells transfected with miR-139-5p mimics, while the activity of the mutant plasmid was not suppressed (Fig. 2B).

### Correlation between the expression of miR-139-5p and Lis1 in PC12 cells

**Figure 3:**
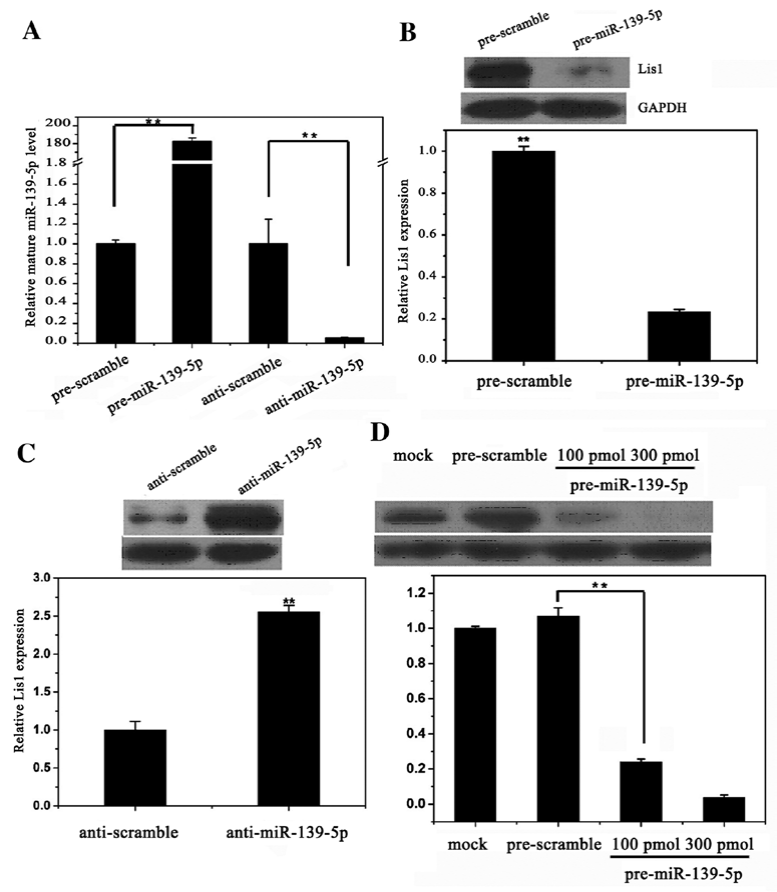
Protein levels of Lis1 as measured by western blot analysis following the induced expression or knockdown of miR-139-5p in PC12 cells. (A) Relative miR-139-5p levels following transfection with pre-miR-139-5p or anti-miR-139-5p. (B) Relative Lis1 protein levels following transfection with pre-miR-139-5p. (C) Relative Lis1 protein levels following transfection with anti-miR-139-5p. (D) Relative Lis1 protein levels following transfection with pre-miR-139-5p at different doses. Data are presented as the means ± standard deviation (SD); ^**^P<0.01.

We confirmed that miR-139-5p was successfully transfected into the PC12 cells (Fig. 3A). There was an inhibitory effect of miR-139-5p on the expression of *Lis1* in the PC12 cells transfected with pre-miR-139-5p after 24 h (Fig. 3B). Transfection with miR-139-5p inhibited the protein expression of Lis1 by approximately 60 and 90% by using 100 and 300 pmol pre-miR-139-5p, respectively (Fig. 3D). By contrast, there was a promotional effect of anti-miR-139-5p on the expression of Lis1 in the PC12 cells transfected with anti-miR-139-5p (Fig. 3C).

### miR-139-5p inhibits cell migration

**Figure 4:**
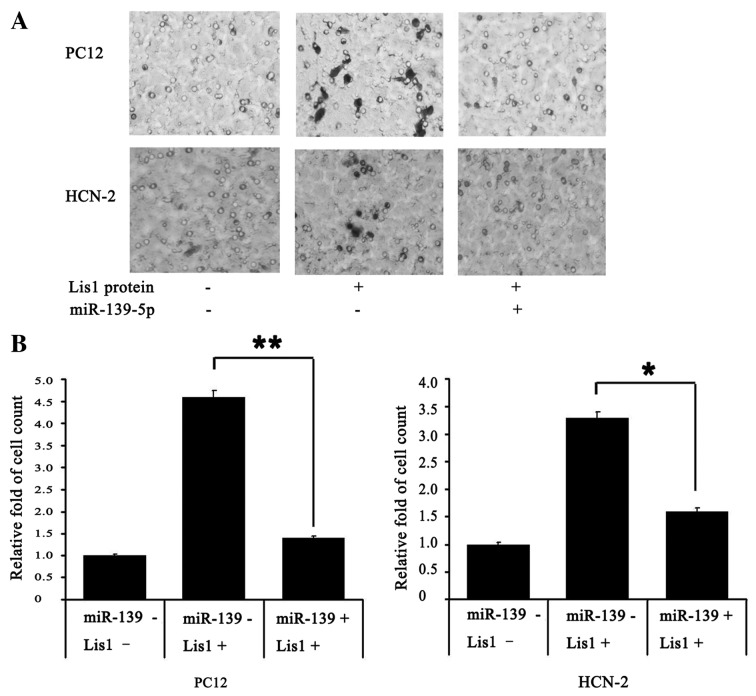
Cell migration experiments in PC12 and HCN-2 cells. (A) Cell migration identified by Transwell experiments in PC12 and HCN-2 cells treated with or without Lis1 protein (magnification, ×200). (B) Relative fold of cell count of cell migration experiments in PC12 and HCN-2 cells. Data are presented as the means ± standard deviation (SD); ^**^P<0.01, ^*^P<0.05.

Transwell assays revealed that miR-139-5p significantly inhibited the migration of PC12 and HCN-2 cells treated with Lis1 protein (Fig. 4A). The migration ability of the PC12 and HCN-2 cells was enhanced only upon the addition of Lis1 protein; this enhanced migration was attenuated by transfection with miR-139-5p.

### miR-139-5p modulates rat brain development by targeting Lis1

**Figure 5:**
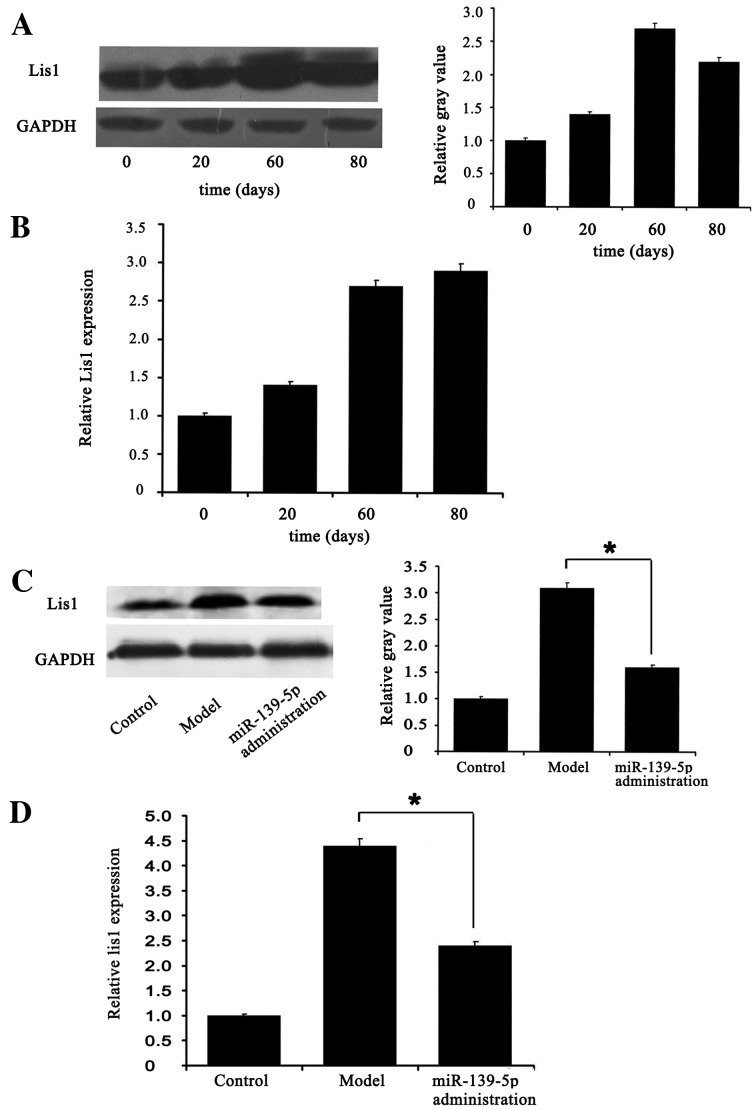
Expression of *Lis1* in immature rats with focal cortical dysplasia induced by liquid nitrogen lesions. (A) Western blot analysis of Lis1 protein levels shown at 0, 20, 60 and 80 days after the rat models were established. (B) qPCR of *Lis1* gene expression shown at 0, 20, 60 and 80 days after the rat models were established. (C) Western blot analysis of Lis1 protein levels showing control (healthy rats), dysplasia model and miR-139-5p administration group 60 days after the rat models were established. (D) qPCR of *Lis1* gene expression showing control, dysplasia model, and miR-139-5p administration group 60 days after the rat models were established. Data are presented as the means ± standard deviation (SD); ^*^P<0.05.

**Figure 6:**
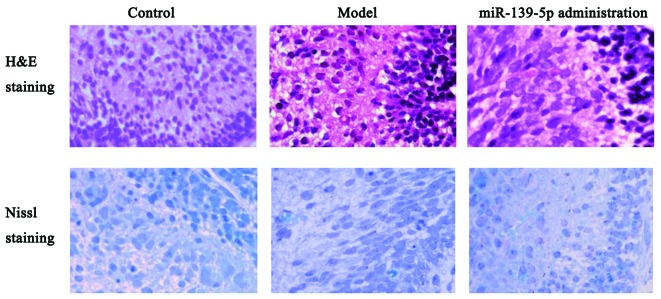
(A) Cell morphology in the control (healthy group), dysplasia model and miR-139-5p administration group shown by hematoxylin and eosin (H&E) staining (magnification, ×200). (B) Cell morphology in the control, dysplasia model and miR-139-5p administration group shown by Nissl staining.

qRT-PCR (Fig. 5B) and western blot analysis (Fig. 5A) indicated that the mRNA and protein levels of *Lis1* were higher at days 20–80 after the rat model of dysplasia was established compared with day 0 (healthy control group). Sixty days after the rat models were established, miR-139-5p was administered, and this led to the marked downregulation of *Lis1* mRNA (Fig. 5D) and protein levels (Fig. 5C). Additionally, it was found that miR-139-5p altered cell morphology in the rat brain, as indicated by H&E and Nissl staining (Fig. 6).

## Discussion

Neuronal migration has been studied extensively for over 30 years in diverse mammalian species from mice to humans (27). Abnormal neuronal migration often leads to cortical dysplasia (28–30). *Lis1* is known to regulate cell proliferation and migration during brain development (31,32) and its expression is known to be disrupted in patients with ILS and MDS, suggesting that a mutation of *Lis1* leads to cortical dysplasia (30). The regulatory function of miRNAs in various developmental, migration and apoptotic pathways of diverse organisms is known. Therefore, the investigation of miRNAs that may regulate *Lis1* expression could potentially lead to the development of novel therapeutic methods for the treatment of patients with cortical dysplasia.

In this study, we profiled the expression of 391 miRNAs in the brains of immature Sprague-Dawley (20–80 days) rats by miRNA microarray to identify the miRNAs responsible for cortical neuronal migration. Among the miRNAs found with significant changes in expression, we selected miR-139-5p to clarify its function in the brain. The screening standard required that the miRNA appear in all databases and have a 2 to 10-fold upregulation in expression, although multiple genes are predicted by several algorithms (TargetScan, miRanda and PicTar) as potential miR-139-5p targets. The microarray results revealed that the expression of miR-139-5p was decreased in the brains of Sprague-Dawley rats (on days 20–80), and that Lis1 is a target of miR-139-5p.

The regulatory function of miRNA in various developmental, differentiation, proliferation, migration and apoptotic pathways of diverse organisms is known (33–35). As expected, we found that miR-139-5p plays a role in rat brain development. Furthermore, miR-139-5p expression was decreased in Sprague-Dawley rats (on days 20–80) with focal cortical dysplasia induced by liquid nitrogen lesions. Due to the significant change in miR-139-5p expression during rat brain development, our data suggest that *Lis1* is a target for miR-139-5p. As *Lis1* is associated with neuronal migration, we speculate that miR-139-5p regulates rat cortical neuronal migration by modulating the expression of certain target genes. These targets, as predicted by multiple algorithms (36), include *Lis1*, *Capn8*, *Gmfb*, *Mapk1*, *Dclk1*, *Vim*, *Mgst1*, *Gnb1*, *Klf15* and *Cdkn1b* (37).

Studies have previously demonstrated the upregulated expression of *Lis1* by proteomic analysis of a freeze-lesion model of focal cortical dysplasia (38,39). Furthermore, *Lis1* is a key gene in brain development due to its role in cell proliferation and migration (40,41). Notably, in our study, *Lis1* expression was markedly upregulated at day 20 and its expression was maintained at these high levels until day 80 (Fig. 5A and B). This is likely an important reflection of the critical role of *Lis1* in promoting cell proliferation and migration. Cell migration assays revealed that miR-139-5p significantly inhibited the migration of PC12 and HCN-2 cells treated with or without Lis1 protein. At the same time, miR-139-5p was administered to the rats with focal cortical dysplasia; miR-139-5p administration markedly decreased the expression of *Lis1*. In addition, the injured cortex of these rat models showed a certain degree of recovery. Furthermore, we confirmed that miR-139-5p targets *Lis1* and inhibits its expression, as confirmed by luciferase assay.

It can be concluded that the upregulation of Lis1 in the rat brain is at least partially caused by a reduction in miR-139-5p. As the upregulation of Lis1 can promote the occurrence of certain developmental events in the rat brain, miR-139-5p modulates rat brain development thorugh the regulation of Lis1 expression. miR-139-5p may also contribute to rat brain development by affecting the expression of several other putative targets (30,42,43). Futher studies are required to validate other predicted targets involved in brain development. The data presented in our study suggest an important role of miR-139-5p in regulating cell migration, thus offering a novel target for the development of therapeutic agents against focal cortical dysplasia.

## Abbreviations

UTR: untranslated region
qRT-PCR: quantitative reverse transcription-polymerase chain reaction
FBS: fetal bovine serum
SPF: specific pathogen-free
PBS: phosphate-buffered saline
